# Impact of Geocoding Methods on Associations between Long-term Exposure to Urban Air Pollution and Lung Function

**DOI:** 10.1289/ehp.1206016

**Published:** 2013-07-03

**Authors:** Bénédicte Jacquemin, Johanna Lepeule, Anne Boudier, Caroline Arnould, Meriem Benmerad, Claire Chappaz, Joane Ferran, Francine Kauffmann, Xavier Morelli, Isabelle Pin, Christophe Pison, Isabelle Rios, Sofia Temam, Nino Künzli, Rémy Slama, Valérie Siroux

**Affiliations:** 1 Inserm (Institut National de la Santé et de la Recherche Médicale), CESP (Centre de recherche en Épidémiologie et Santé des Populations), U1018, Respiratory and Environmental Epidemiology Team, Villejuif, France; 2 Université Paris Sud 11, UMRS 1018, Villejuif, France; 3 Inserm, U823, Institut Albert Bonniot, Team of Environmental Epidemiology Applied to Reproduction and Respiratory Health, Grenoble, France; 4 Université Joseph Fourier, Grenoble, France; 5 Air Rhône-Alpes, Grenoble, France; 6 Department of Pediatrics, CHU (Centre Hospitalier Universitaire), Grenoble, France; 7 Inserm, U1055, Grenoble, France; 8 PCMAC (Pôle Cancérologie, Médecine Aiguë et Communautaire), CHU, Grenoble, France; 9 Swiss Tropical and Public Health Institute, Basel, Switzerland; 10 University of Basel, Basel, Switzerland

## Abstract

Background: Errors in address geocodes may affect estimates of the effects of air pollution on health.

Objective: We investigated the impact of four geocoding techniques on the association between urban air pollution estimated with a fine-scale (10 m × 10 m) dispersion model and lung function in adults.

Methods: We measured forced expiratory volume in 1 sec (FEV_1_) and forced vital capacity (FVC) in 354 adult residents of Grenoble, France, who were participants in two well-characterized studies, the Epidemiological Study on the Genetics and Environment on Asthma (EGEA) and the European Community Respiratory Health Survey (ECRHS). Home addresses were geocoded using individual building matching as the reference approach and three spatial interpolation approaches. We used a dispersion model to estimate mean PM_10_ and nitrogen dioxide concentrations at each participant’s address during the 12 months preceding their lung function measurements. Associations between exposures and lung function parameters were adjusted for individual confounders and same-day exposure to air pollutants. The geocoding techniques were compared with regard to geographical distances between coordinates, exposure estimates, and associations between the estimated exposures and health effects.

Results: Median distances between coordinates estimated using the building matching and the three interpolation techniques were 26.4, 27.9, and 35.6 m. Compared with exposure estimates based on building matching, PM_10_ concentrations based on the three interpolation techniques tended to be overestimated. When building matching was used to estimate exposures, a one-interquartile range increase in PM_10_ (3.0 μg/m^3^) was associated with a 3.72-point decrease in FVC% predicted (95% CI: –0.56, –6.88) and a 3.86-point decrease in FEV_1_% predicted (95% CI: –0.14, –3.24). The magnitude of associations decreased when other geocoding approaches were used [e.g., for FVC% predicted –2.81 (95% CI: –0.26, –5.35) using NavTEQ, or 2.08 (95% CI –4.63, 0.47, *p* = 0.11) using Google Maps].

Conclusions: Our findings suggest that the choice of geocoding technique may influence estimated health effects when air pollution exposures are estimated using a fine-scale exposure model.

Citation: Jacquemin B, Lepeule J, Boudier A, Arnould C, Benmerad M, Chappaz C, Ferran J, Kauffmann F, Morelli X, Pin I, Pison C, Rios I, Temam S, Künzli N, Slama R, Siroux V. 2013. Impact of geocoding methods on associations between long-term exposure to urban air pollution and lung function. Environ Health Perspect 121:1054–1060; http://dx.doi.org/10.1289/ehp.1206016

## Introduction

Epidemiological studies have reported associations between daily variations in air pollutants and short-term changes in lung function (Brunekreef et al. 1995; McCreanor et al. 2007). Fewer studies have estimated effects of long-term air pollutant exposures on the level, growth, or decline of pulmonary function. Many of these studies have used central monitoring station data as a proxy measure of exposure for all residents of a community; thus, health effect estimates are based on between-community comparisons (Gotschi et al. 2008b). However, because air pollution concentrations are highly variable within communities, this approach is prone to exposure misclassification. The most common approaches for estimating long-term air pollution exposures at the individual level are based on concentrations of outdoor air pollutants estimated at each individual’s home address. In the last decade, several methods have been developed to better estimate local spatial distribution of exposure to air pollution, such as dispersion models (Jerrett et al. 2005). These exposure models require that home addresses be geocoded (i.e., assigned geographic coordinates with latitude and longitude) to link each address with a spatial estimate of exposure. Previous studies assessed the positional error of different geocoding techniques and concluded that geocoding of addresses is generally accurate (Bonner et al. 2003; Duncan et al. 2011). However, when using models with fine-scale spatial resolution to estimate exposure to pollutants with high spatial variation, minimal errors in geographical coordinates of home addresses may lead to large differences in exposure estimates, and consequently to biased risk estimates. To our knowledge, no study has investigated the impact of geocoding errors on estimated health effects of traffic-related air pollutants.

We aimed to investigate the impact of the geocoding technique error when assigning home outdoor exposure on the association between long-term exposure to urban air pollution, estimated using a fine-scale dispersion model, and lung function in adults.

## Methods

*Description of the populations.* Participants included in the analysis were part of two large, well-characterized epidemiological studies. The Epidemiological Study on the Genetics and Environment on Asthma (EGEA) is a French cohort of 2,047 participants (asthma patients enrolled from hospital chest clinics, their first-degree relatives, and controls who were recruited from other hospital wards or from electoral lists) enrolled between 1991–1995 from five French cities. Subjects were followed up between 2003 and 2007 (Kauffmann et al. 1997, 1999; Siroux et al. 2009). The European Community Respiratory Health Survey (ECRHS) is a population-based cohort of young adults, enriched with participants with respiratory symptoms, recruited from 1991 to 1993 in 28 western European cities and followed up between 1999 and 2001 (Burney et al. 1994; European Community Respiratory Health Survey II Steering Committee 2002). A detailed standardized questionnaire on respiratory health was used in both studies. Participants in these studies have been extensively characterized with regard to their respiratory health and risk factors using standardized protocols and questionnaires. We focus here on participants living in Grenoble, a city in southeastern France with a population of 496,951 (French National Institute for Statistics and Economic Studies 2010).

The present cross-sectional study includes 354 study participants living in Grenoble (164 from EGEA2, and 190 from ECRHSII) with complete lung function data and a valid postal address that was geocoded precisely using all four techniques (see Supplemental Material, Figure S1).

Ethical approval to perform the study was obtained for both surveys from the relevant committees (Hôpital Necker–Enfants Malades, Paris, France, for EGEA2; Comité de Protection des Personnes Participant à la Recherche Biomédicale de Bichat-Claude-Bernard, Paris, France, for ECRHSII France).

*Outcomes.* Spirometry was performed according to similar standardized protocols in both studies. EGEA and ECRHS participants were examined in the same medical center by the same technician. The main difference was that a water-sealed spirometer (BAIRES system; Biomedin, Padua, Italy) was used in ECRHSII, and a flow–volume spirometer (SpiroDyn’R; SAS Dyn’R, Aix-en-Provence, France) in EGEA2. In both studies, standardized operating procedures were implemented and controlled, including calibration of all equipment before each measurement, and quality control visits were performed throughout both studies by study coordinators to ensure correct protocols by field staff. Participants were asked to perform three to nine attempts to provide at least three technically acceptable spirometry maneuvers. Both forced expiratory volume in 1 sec (FEV_1_) and forced vital capacity (FVC) were expressed in percent predicted value and were computed using the sex- and age-specific equations from Stanojevic et al. (2008).

Asthma was defined by a positive response to “Have you ever had asthma?” in ECRHS. In EGEA, it was defined by a positive response to “Have you ever had attacks of breathlessness at rest with wheezing?” or “Have you ever had asthma attacks?” or by being recruited as an asthma patient in chest clinics.

Allergic sensitization was defined by skin-prick tests to 11 allergens in EGEA (cat, *Dermatophagoides pteronyssinus*, *Blattela germanica*, olive, birch, *Parieteria judaica*, timothy grass, ragweed pollen, *Aspergillus*, *Cladosporium herbarum*, *Alternaria tenuis*) and by specific IgE to four allergens in ECRHS (cat, *Dermatophagoides pteronyssinus*, *Cladosporium,* and timothy grass).

*Geocoding.* The exact home addresses were geocoded using building matching in addition to three methods based on spatial interpolation.

Building matching. Each home address was manually geocoded using free on-line French cadastral maps (Ministère de l’Économie et des Finances 2012) to determine the coordinates of the approximate center of the building. The French cadastral plan is an administrative database that contains all digitalized maps developed by land surveyors for all landed properties in France.

Spatial interpolation methods. Interpolation techniques assign each address to an address-ranged street segment that is georeferenced within a streetline database and interpolate the address position along the segment (Zimmerman et al. 2007). Specifically, default settings for the following techniques were used:

NavTEQ: The streets network of NavTEQ® software automatically geocodes each address by spatial interpolation along street axis, with each address assigned coordinates corresponding to 15 m to the right or left (depending on whether the street number is even or odd) of the axis running down the middle of the street. NavTEQ geocoding was performed by a commercial geocoding company.

Google Maps: A free Internet service developed using the Google Maps® (https://maps.google.com/) Internet mapping application programming interface (API) was used to automatically assign coordinates corresponding (in theory) to the location of the building or parcel entrance on the street (Muraz 2007).

Multimap: The free Internet service Multimap® was used to manually assign coordinates for the street entrance of the building or parcel for each address. In 2010 Multimap was bought by Bing Maps® (http://www.bing.com/maps/) (Microsoft Corp., Redmond, WA, USA), and now it also geocodes automatically (Microsoft Corp. 2013).

“Manually assigned coordinates” means that each address was looked at individually; whereas “automatically assigned coordinates” refers to a geocoding batch.

The building-matching technique was selected as the reference method *a priori* because it does not rely on spatial interpolation, which can result in positional errors because it assumes that addresses are evenly distributed along a street segment (Hay et al. 2009) and does not take into account the exact width of the street. To support the use of the building-matching method as the reference, we compared coordinates estimated using the four methods to global positioning system (GPS) coordinates (from a Garmin Dakota 20 GPS system; Garmin Inc., Southampton, UK) for 42 addresses randomly selected among the 450 addresses in our study. As expected, the positional error was lower for coordinates based on building matching [median distance (25th–75th centiles) from GPS coordinates of 13.8 m (range, 10.3–18.9 m)] than for the spatial interpolation techniques [28.8 m (13.9–57.1 m) for NavTEQ, 21.4 m (8.9–46.7 m) for Google maps, and 36.4 m (16.7–76.5 m) for Multimap].

The present study was limited to 354 participants at addresses that were precisely geocoded using all four techniques. Geocoding was defined as “precise” *a*) if the building was found without doubt for the building-matching technique (*n* = 429, 95.3% of 450 addresses evaluated); *b*) if the exact address was found automatically with the highest possible precision (< 15 m) using NavTEQ (*n* = 387, 86.0%); *c*) if the address was found automatically using GoogleMaps (code 8) (*n* = 425, 94.4%); or, *d*) for Multimap, if there was a high correspondence between the original address and the location text given by the website and only one set of coordinates was proposed for the address (*n* = 410, 91.1%). The main reasons addresses could not be precisely geocoded were that the street number was missing or did not exist, or that the street name was misspelled.

*Air pollution exposure.* Annual concentrations of nitrogen dioxide (NO_2_) and particulate matter with an aerodynamic diameter of ≤ 10 µm (PM_10_) at the home addresses were estimated using the SIRANE dispersion model developed on a 10 m × 10 m grid for 2004 for NO_2_, and 2008 for PM_10_ (Soulhac et al. 2011; see also Supplemental Material, Figure S2). These yearly averages were combined with time-specific measures to capture temporal variations in exposure using a previously described approach (Lepeule et al. 2010; Slama et al. 2007). The time-specific measures were obtained from a permanent background monitor that operated continuously during the study period (Villeneuve les Frênes monitor, Grenoble) (see Supplemental Material, Figure S3). The exposure window used for our primary analyses was the 12-month period before the lung function measurement. In addition, we estimated short-term exposures on the day of the lung function measurement (i.e., lag 0) using concentrations measured by the same background monitor that were considered to be representative of the air quality for the city of Grenoble as a whole.

*Statistical analysis.* For each address, the distances between the coordinates assigned by each geocoding technique were calculated. Pearson’s correlations and Bland–Altman plots were generated to assess the agreement between air pollution concentrations obtained with each spatial interpolation technique and the building-matching method.

Associations between lung function and average long-term exposure (during the previous 12 months) to NO_2_ or PM_10_ were estimated using linear regression models that used general estimating equations to account for the family structure (nonindependent observations) of the EGEA study population. We confirmed that both FEV_1_ and FVC were normally distributed based on a homoscedasticity test and by evaluating the distributions of the model residuals (data not shown). Three models were conducted for each exposure (i.e., NO_2_ and PM_10_ separately), beginning with unadjusted single pollutant models of average concentrations during the previous 12 months (model 0). Next we adjusted for basic covariates [sex, age, body mass index (BMI), active smoking, environmental tobacco smoke (ETS), occupational group, use of inhaled corticosteroids, atopy, asthma, and study (model 1)]. Finally, we also included exposure to the same pollutant at lag 0 to adjust for any short-term effects of exposure (model 2). Exposures were coded as continuous variables, and associations are reported for a one-interquartile range (1-IQR) increase in exposure (5.2 μg/m^3^ for NO_2_ and 3.0 μg/m^3^ for PM_10_). We used model 2 results to compare associations with exposures estimated using the four different geocoding techniques. In addition to estimating associations for the study population as a whole, we stratified analyses by asthma status and by study.

We performed two sensitivity analyses to evaluate the influence of back extrapolating SIRANE model estimates to earlier periods. First, we estimated associations with NO_2_ exposures estimated by the SIRANE 2004 model, and with PM_10_ estimates from the SIRANE 2008 model, instead of back extrapolating the SIRANE model estimates to the specific 12-month period before each participant’s lung function measurement. In addition, we estimated associations with exposures averaged over the 12 months before and the 12 months after lung function testing (24-month average) to assess the impact of the back extrapolation.

## Results

*Description of the population.* Mean (± SE) FEV_1_ and FVC% predicted were 100.1 ± 15.1 and 102.2 ± 13.7, respectively (Table 1). The mean age was 45.6 years, and 48.6% of participants were female. Of the 354 participants included in the analysis, 93 were classified as ever having had asthma based on self-report or recruitment from an asthma clinic (Table 1). Participants were distributed throughout the urban area, without any obvious geographical clusters (see Supplemental Material, Figure S3).

**Table 1:** Description of the study population [mean ± SE for continuous variables or *n* (%) for categorical variables].

Characteristic	*n*	All (*n*=354)	Participants without asthma (*n*=261)	Participants with asthma (*n*=93)	*p*-Value^*a*^
General characteristics
Age	354	45.6±13.3	46.9±12.6	41.9±14.5	0.001
Sex, female	354	172 (48.6)	130 (49.8)	42 (45.2)	0.44
BMI (kg/m^2^)	354	23.9±3.8	23.7±3.6	24.4±4.5	0.15
Occupational group	334				0.06
Manager		140 (41.9)	112 (44.8)	28 (33.3)
Technician		140 (42.5)	97 (38.8)	45 (53.6)
Manual worker		52 (15.6)	41 (16.4)	11 (13.1)
Active smoking	353				0.54
Nonsmoker		158 (44.8)	114 (43.8)	44 (47.3)
Former smoker		103 (29.2)	80 (30.8)	23 (24.7)
Current smoker		92 (26.1)	66 (25.4)	26 (28.0)
ETS	354	171 (48.3)	123 (47.1)	48 (51.6)	0.46
Atopy, yes	342	145 (42.4)	73 (29.0)	72 (80.0)	<0.0001
Use of inhaled corticosteroids	353	36 (10.2)	4 (1.5)	32 (34.4)	—
Study
EGEA	354	164 (46.3)	108 (41.4)	56 (60.2)	0.002
ECRHS	354	190 (53.7)	153 (58.6)	37 (39.8)
Lung function
FEV_1_% predicted^*b*^	354	100.1±15.1	102.1±13.8	94.3±17.0	0.0001
FVC% predicted^*b*^	354	102.2±13.7	102.2±13.7	102.3±13.6	0.97
FEV_1_/FVC% predicted^*b*^	354	97.7±9.4	99.7±7.1	92.0±12.4	<0.0001
^***a***^*p*-Value comparing participants with and without asthma, by χ^2^ for categorical variables, and by *t*-test for age and BMI. ^***b***^Using the predicted equations from Stanojevic etal. (2008).					

*Positional error between the geocoding techniques.* Median distances between coordinates estimated using the building-matching method and the spatial interpolation techniques (NavTEQ, Google Maps, and Multimap) were 27.9 m, 26.4 m, and 35.6 m, respectively. The shortest median distance was between Multimap and NavTEQ geocodes, and the longest median distance was between Multimap and the building-matching technique (Table 2).

**Table 2:** Median (25th–75th percentiles) distance (m) between the home addresses estimated by the different geo­coding techniques (*n* = 354).

Geocoding technique	NavTEQ	Google Maps	Multimap
Building matching	27.9 (13.7–54.7)	26.4 (12.9–55.0)	35.6 (19.7–78.0)
NavTEQ	—	24.7 (11.8–59.4)	18.9 (12.6–66.9)
Google Maps	—	—	21.8 (8.9–65.4)
Multimap	—	—	—

**Figure 1:**
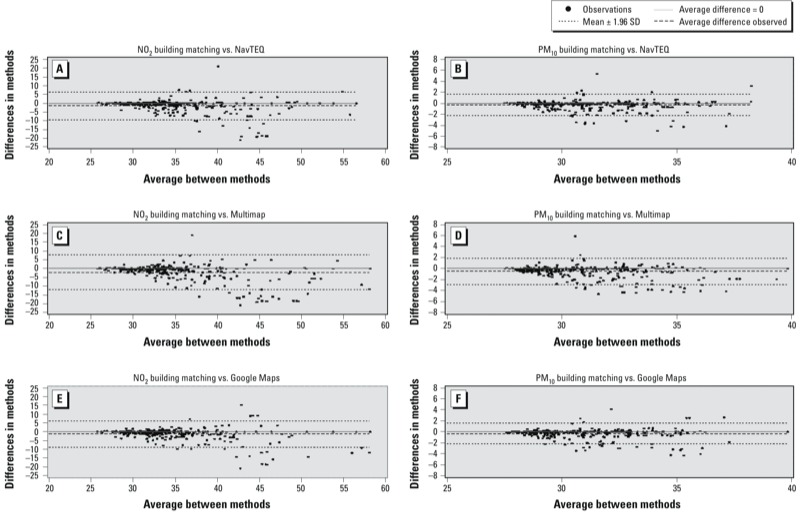
Bland–Altman plots comparing the NO_2_ (*A,C,E*) and PM_10_ (*B,D,F*) concentrations estimated using the building-matching geocoding technique to the pollutant concentrations estimated using the spatial interpolation geocoding techniques [NavTEQ (*A,B*), Multimap (*C,D*), and Google Maps (*E,F*)].

*Air pollutant exposures.* Median annual NO_2_ and PM_10_ concentrations were 33.0 μg/m^3^ and 30.5 μg/m^3^, respectively, as assessed at coordinates defined using the building-matching technique. When using the other geocoding techniques, estimated concentrations were slightly higher (Table 3). Median differences in exposure between the building-matching geocodes and the spatial interpolation geocodes were –0.07 μg/m^3^ (NavTEQ), –0.06 (Google Maps), and –0.15 μg/m^3^ (Multimap) for NO_2_ and –0.02, –0.02, and –0.06 μg/m^3^ for PM_10_, respectively (see Supplemental Material, Table S1). The funnel shape of the Bland–Altman plots showed that the three interpolation techniques tended to overestimate the air pollution concentrations compared with the building matching, particularly for higher values, supporting a multiplicative structure of the errors (Figure 1). Mean annual pollutants concentrations were highly correlated across the various geocoding techniques (*r* ≥ 0.75 for NO_2_ and *r* ≥ 0.89 for PM_10_) (see Supplemental Material, Table S2).

**Table 3:** Air pollutant concentrations (annual mean) according to geo­coding technique.

Air pollutant geo­coding technique	Minimum	25th percentile	Median	50th percentile	Maximum
NO_2_
Building matching	25.7	30.7	33	35.9	58.2
NavTEQ	25.7	31.2	33.7	37.8	59
Google Maps	25.7	31.1	33.5	37.2	64
Multimap	25.7	31.2	33.6	38.5	64
PM_10_
Building matching	27.5	29.1	30.5	32.4	39.8
NavTEQ	27.5	29.3	30.7	32.6	39.2
Google Maps	27.6	29.3	30.6	32.6	39.8
Multimap	27.5	29.3	30.8	32.8	40.3

Compared with ECRHS, levels of NO_2_ and PM_10_ and contrasts of PM_10_ exposures were higher in EGEA (see Supplemental Material, Table S3), which also included more participants with asthma (34% compared with 19.5%). On average, compared with those without asthma, participants with asthma tended to be exposed to slightly higher levels of NO_2_ and PM_10_ during the 12 months before the examination (see Supplemental Material, Table S4). However, exposures were comparable according to asthma status when each study was evaluated separately (data not shown). NO_2_ and PM_10_ lag 0 concentrations were not correlated with annual concentrations (Spearman *r =* 0.005 and 0.13, respectively).

*Association between lung function and exposures estimated using building matching.* There were no statistically significant crude associations of FEV_1_ or FVC with annual or lag 0 concentrations of NO_2_ or PM_10_ estimated for addresses geocoded using the building-matching method (*p*-values > 0.16) (model 0; see Supplemental Material, Table S5). After adjustment for potential individual confounders (model 1), IQR increases in average exposures during the previous 12 months were associated with lower FEV_1_ (β = –1.65; 95% CI: –3.34, 0.04 and β = –3.95; 95% CI: –7.09, –0.81, for NO_2_ and PM_10_, respectively) and FVC (β = –1.71; 95% CI: –3.26, –0.16 and β = –3.99; 95% CI: –6.87, –1.11, for NO_2_ and PM_10_, respectively) (see Supplemental Material, Table S5). Further adjustment for lag 0 concentrations had no impact on the estimates associated with annual level (see Supplemental Material, Table S5). Associations between PM_10_ and both FEV_1_ and FVC tended to be stronger in participants with asthma compared with those without asthma (see Supplemental Material, Table S6) and in EGEA versus ECRHS participants (see Supplemental Material, Table S7).

Associations with exposures estimated without back extrapolation were closer to the null and no longer statistically significant, whereas associations with the 24-month average exposures (12 months before and 12 months after the lung function measurements) were more similar but less statistically significant than associations with 12-month average exposures (see Supplemental Material Table S8).

**Figure 2:**
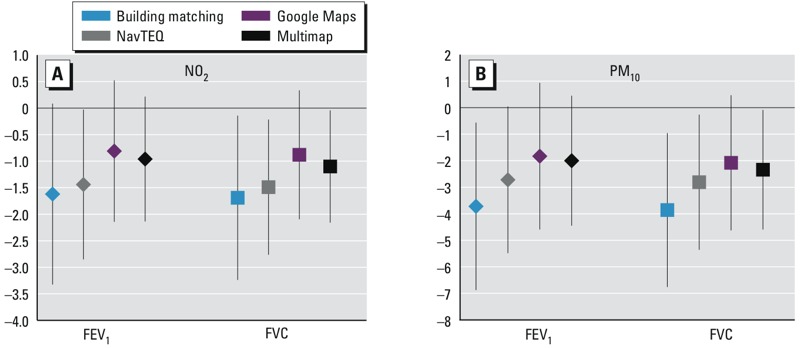
Adjusted associations of FEV_1_ and FVC with a 1-IQR increase in average residential NO_2_ [5.2 μg/m^3^ (*A*)] or PM_10_ [3.0 μg/m^3^ (*B*)] during the 12 months before lung function testing, according to the technique used to geocode home addresses. Models were adjusted for sex, age, BMI, active smoking, ETS, occupational group, atopy, study and pollutant concentration on the day of examination (*n* = 310 and 316 for NO_2_ and PM_10_, respectively).

*Impact of geocoding technique on estimated air pollution effects.* For both pollutants, associations with exposures estimated using building matching to geocoded addresses were stronger and had smaller *p*-values than associations with exposures estimated using spatial interpolation techniques for geocoding (Figure 2). For example, the model 2 coefficient for FVC in association with a 1-IQR increase in PM_10_ was –3.86 (95% CI: –0.96, –6.76, *p* = 0.01) when building matching was used for geocoding compared with –2.81 (95% CI: –0.26, –5.35, *p* = 0.03) when NavTEQ was used or –2.08 (95% CI –4.63, 0.47, *p* = 0.11) when Google Maps was used.

## Discussion

Using a high spatially resolved dispersion model to assess home outdoor NO_2_ and PM_10_ exposure, we showed for the first time that the geocoding techniques used to individually assign estimates of long-term exposure may have an impact on the estimated associations with lung function.

The strengths of this analysis relate to the fact that we relied on a rather homogeneous population with all individuals living in a restricted geographical urban area, with well-characterized respiratory health using standardized lung function measurements. A further strength relates to the very fine spatial resolution of the exposure model. We focused on NO_2_ and PM_10_ in ambient air at the home address as proxy measures of individual exposure to traffic-related emissions. As usually done in epidemiological studies of air pollution effects on health, air pollution exposures were estimated at the home address, and exposures at other locations were not accounted for in our analysis.

In contrast with most previous studies, we used data from permanent air quality monitors for the 12 months prior to lung function measurements to capture time trends in air pollutants, instead of using the average exposure during the calendar year of the examination, which would include periods before and after the outcome measurement. Both approaches are limited by uncertainties regarding the exact biologically relevant exposure window. Moreover, the use of data from a permanent monitoring station to back extrapolate exposures to the 12 months before each participant’s lung function measurements assumed that temporal trends in air pollution levels were homogeneous across the study area, which may not be true if strong urban changes took place in the study period. A study in Oslo, Norway, showed that fine spatially resolved air pollution levels estimated by land-use regression models developed from NO_x_ (nitrous oxides) measurements conducted 3 years apart were quite strongly correlated (Madsen et al. 2011). In our study, the back or forward extrapolation was performed over relatively short periods of ≤ 4 years for NO_2_ and ≤ 8 years for PM_10_. Weaker associations with air pollution exposure estimated without using back extrapolation (i.e., based on average annual exposures to NO_2_ and PM_10_ at home addresses in 2004 or 2008) or for average exposures over 24 months (including the 12 months before and the 12 months after lung function measurements) suggest that our *a priori*–chosen 12-month exposure window before the examination may be closer to the biologically relevant period than the exposure windows including periods after the examination. Our finding that effect estimates for exposures derived from the 24-month back extrapolated model were intermediate between associations with exposures based on the nonextrapolated model and the 12-month extrapolated model supports this hypothesis.

Our findings add to the existing literature, suggesting a role of chronic exposure to outdoor air pollution on lung function in adults. The SAPALDIA (Swiss Study on Air Pollution and Lung Disease in Adults) study first showed in a cross-sectional analysis that individually assigned SO_2_, NO_2_, and PM_10_ yearly levels were associated with lower lung function parameters (Ackermann-Liebrich et al. 1997) and that improvement in ambient PM_10_ was associated with an attenuated decline in lung function (Downs et al. 2007). A British study also reported that chronic exposure to PM_10_, NO_2_, and SO_2_, was associated with reduced FEV_1_ in adults (Forbes et al. 2009). Other studies have reported stronger associations between lung function and gases than between lung function and PM, but these associations are not consistent nor conclusive (Gotschi et al. 2008a). Moreover, most of these studies have focused on between-city comparisons of ambient exposures measured using a single monitor, whereas we used a high-resolution model to capture spatial variation due to traffic-related near-road pollution. Thus, comparison with the vast majority of lung function studies may not be appropriate (Gotschi et al. 2008a).

We found a trend for stronger associations between PM_10_ exposures and lung function among EGEA versus ECHRS study participants, which could partly be explained by higher level and contrast of PM_10_ exposure in EGEA compared with ECRHS (median 12-month average concentration of 32.1 µg/m^3^, IQR 31.0–33.2 µg/m^3^ for EGEA vs. median 29.1 µg/m^3^, IQR 28.6–29.8 µg/m^3^ for ECHRS). There is also a potential for greater misclassification error when back extrapolating PM_10_ concentrations based on the 2008 SIRANE model to 12-month time windows before lung function testing in ECRHSII participants (conducted between 1999–2001) compared with EGEA (conducted between 2003 and 2007). In addition, ECRHS was a population-based study enriched with participants with asthma, whereas EGEA was a cohort study that enrolled asthma patients recruited in chest clinics, their first-degree relatives, and controls. However, participants from both studies were evenly distributed within the study area, and were geocoded by the same person using the same protocols. Therefore, differences between the two populations are not expected to affect differences in associations when different geocoding methods are used to estimate exposures.

A substantial body of literature exists on the positional accuracy of geocoded addresses using the spatial interpolation techniques (Bonner et al. 2003; Cayo and Talbot 2003; Duncan et al. 2011; McElroy et al. 2003; Zandbergen et al. 2012). It has been shown that the magnitude of the positional errors using such techniques varies according to the street lengths, with wide within-city variations even within the same city, and between urban and rural areas, with more accurate geocodes in urban areas compared with rural areas (Cayo and Talbot 2003; Hay et al. 2009). The urban-rural variation was not much of a concern in our study because all participants lived in an urban area.

The degree to which the positional errors in geocoding affect the exposure assessment and the health risk assessment depends on the spatial resolution of the exposure model. Our analysis illustrates the impact of geocoding errors on exposure estimates derived from a highly resolved dispersion model. In our study, residential outdoor exposure estimates based on spatial interpolation techniques were higher than exposures estimated when building matching was used to geocode home addresses. This may be explained by systematic differences in the location of the coordinates, which are expected to be closer to the street (and thus closer to the traffic and more exposed) when spatial interpolation techniques are used compared with the building-matching method, which locates address coordinates at the center of the building or the parcel. In addition, when spatial interpolation is used, the location of a home is estimated proportionally to the length of the street segment (e.g., number 51 is assumed to be located in the middle of the street segment if building numbers range from 1 to 100), whereas the building-matching technique relies on information about the exact location of each street address.

The funnel shape of the Bland–Altman plots comparing NO_2_ and PM_10_ exposure estimates based the spatial interpolation methods with estimates derived using the building-matching technique (which is assumed to be the most accurate) indicates a multiplicative error structure, such that higher exposures are estimated with greater error. This observation is consistent with previous findings that indicated that street geocoding overestimates the number of children potentially exposed to traffic-related air pollutants compared with estimates derived using building matching (Zandbergen and Green 2007). Such errors are expected to bias health effect estimates toward the null, consistent with our findings of weaker associations when exposures are derived using spatial interpolation to geocoded addresses.

Our findings suggest that in urban settings, spatial interpolation techniques for address geocoding may lead to underestimated effects of air pollution on health outcomes when using any spatially resolved exposure model, including dispersion models (as in our study), land-use regression, or satellite-based exposure models. The narrower CIs of the parameters corresponding to air pollution levels assessed with the spatial interpolation geocodes may be explained by the larger variance of exposure when using this geocoding approach. Indeed the variance of the linear regression coefficient is known to be inversely related to the variance of the corresponding predictor (Kleinbaum et al. 1998). However, our results do not support findings from a simulation study, which indicated that the strength of the exposure–disease association remained stable when different geocoding techniques were used and that the precision of effect estimates generally increased as the quality of the geocoding decreased (Mazumdar et al. 2008). We recommend caution in the extrapolation of our conclusions to other regions and cities. First, the availability and accuracy of different geocoding techniques may vary among countries, as well as the resolution of the exposure model (upon which the impact of geocoding errors on exposures will depend). Second, our study was restricted to an urban setting, and the amplitude of geocoding errors, and their impact on exposure estimates, may be different in more rural areas. More studies are needed in other types of areas and countries.

The fact that differences among techniques were greater for NO_2_ than PM_10_ concentrations was expected because spatial variation is more pronounced for NO_2_ than PM_10_ (Krzyzanowski et al. 2005), and small errors in geocoding would therefore be expected to lead to greater differences in air pollution exposure estimates, for example, in near-traffic situations where NO_2_ levels strongly vary. Thus, we expected that less precise geocoding would have a greater impact on health effect estimates for NO_2_ than PM_10_. However, it was not possible to formally test this hypothesis given that associations between NO_2_ exposure and lung function were borderline or null in our study population. Finally, we did not have information to correct exposure estimates for differences in the vertical elevation of individual residences, which may be located on the upper floors in multifloor apartment buildings.

## Conclusion

Our results suggest that the choice of geocoding technique could have an impact on health effect estimates when high-resolution exposure models are used to capture within-city variability. This is an issue to carefully consider because address geocoding and fine spatial scale exposure models are increasingly used in epidemiological studies.

## Supplemental Material

(602 KB) PDF
