# Supplementary material for: Impact of Geocoding Methods on Associations between Long-term Exposure to Urban Air Pollution and Lung Function
Source: Environ Health Perspect. 2013 Jul 3;121(9):1054–60. doi: 10.1289/ehp.1206016 (PMC3764075; doi:10.1289/ehp.1206016)
Supplement: (602 KB) PDF [file ehp.1206016.s001.pdf]

Environmental Health Perspectives doi:10.1289/ehp.1206016

SUPPLEMENTAL MATERIAL

Impact of Geocoding Methods on Associations between Long-term Exposure to Urban Air Pollution and Lung Function

Bénédicte Jacquemin, Johanna Lepeule, Anne Boudier, Caroline Arnould, Meriem Benmerad, Claire Chappaz, Joane Ferran, Francine Kauffmann, Xavier Morelli, Isabelle Pin, Christophe Pison, Isabelle Rios, Sofia Temam, Nino Künzli, Rémy Slama, and Valérie Siroux.

Table of contents:

Page 2 Table S1: Median (min, 25th centile, 75th centile, max) of the mean annual pollutant concentration (µg/m3) differences between the building matching geocodes and each spatial interpolation geocodes, n=354

Page 2 Table S2: Correlation (Spearman) between the pollutant concentrations according to the different geocoding techniques, n=354

Page 3 Table S3: Median (minimum, 25th centile, 75th centile and maximum) annual pollutant concentrations using the building matching geocoding technique by survey (n=354)

Page 3 Table S4: Median annual (minimum, 25th centile, 75th centile and maximum) pollutant concentrations using the building matching geocoding technique by asthma status (n=354)

Page 4 Table S5: Associations between air pollution and lung function per one increase of one interquartile range in air pollutants levels (5.2 µg/m3 for NO2 and 3.0 µg/m3 for PM10) estimated using matching building.

Page 5 Table S6: Associations between air pollution and lung function per one increase of one interquartile range in air pollutants levels (5.2 µg/m3 for NO2 and 3.0 µg/m3 for PM10) stratified by asthma status.

Page 6 Table S7: Associations between air pollution and lung function per one increase of one interquartile range in air pollutants levels (5.2 µg/m3 for NO2 and 3.0 µg/m3 for PM10) stratified by study.

Page 7 Table S8: Associations between air pollution and lung function per one increase of one interquartile range in air pollutants levels (5.2 µg/m3 for NO2 and 3.0 µg/m3 for PM10), with 12-months back-extrapolated air pollution estimates, without back-extrapolation (as estimated by the SIRANE 2004 model for NO2 and the SIRANE 2008 model for PM10) and with 24-month back-extrapolated air pollution estimates (12 months before and after lung function testing)

Page 8 Figure S1: Description of the study population

Page 9 Figure S2: Mean annual NO2 concentration estimated by the SIRANE dispersion model in 2004 in Grenoble, France

Page 10 Figure S3: Map showing the participant's distribution in the Grenoble area, taking into account the study they come from

Table S1: Median (min, 25th centile, 75th centile, max) of the mean annual pollutant concentration (µg/m^3) differences between the building matching geocodes and each spatial interpolation geocodes, n=354

	A. Building matching versus B.NavTEQ	A. Building matching versus B. Google Maps	A. Building matching versus C.Multimap
**NO_2**			
min	-20.8	-20.8	-20.8
25th centile	-1.7	-1.4	-2.2
median	-0.1	-0.1	-0.2
75th centile	0.1	0.1	0.1
max	21	15.5	19
**PM_{10}**			
min	-5	-4.3	-4.6
25th centile	-0.5	-0.4	-0.7
median	-0.2	0	-0.1
75th centile	0	0	0
max	5.4	4.1	5.9

Table S2: Correlation (Spearman) between the pollutant concentrations according to the different geocoding techniques, n=354

	NO_2			
PM_{10}	A. Building matching	B. NavTEQ	C. Google Maps	D. Multimap
A. Building matching	-	0.76	0.82	0.75
B. NavTEQ	0.89	-	0.82	0.83
C. Google Maps	0.91	0.90	-	0.83
D. Multimap	0.88	0.89	0.91	-

Table S3: Median (minimum, 25th centile, 75th centile and maximum) annual pollutant concentrations using the building matching geocoding technique by survey (n=354)

	NO_2, µg/m^3	PM_{10}, µg/m^3
**ECRHS**		
Min	25.1	27.5
25th centile	29.6	28.6
Median	31.3	29.1
75th centile	34.1	29.8
Max	53.8	34.9
**EGEA**		
Min	27.8	29.5
25th centile	33	31
Median	34.4	32.1
75th centile	37.2	33.2
Max	58.2	39.8
p value (non-parametric test)	<0.001	<0.001

Table S4: Median (minimum, 25th centile, 75th centile and maximum) annual pollutant concentrations using the building matching geocoding technique by asthma status (n=354)

	NO_2, µg/m^3	PM_{10}, µg/m^3
**Among participants with asthma**		
Min	25.7	27.5
25th centile	30.7	29.0
Median	32.9	30.3
75th centile	35.9	32.0
Max	58.2	39.8
**Among participants without asthma**		
Min	26.7	27.7
25th centile	31.3	29.7
Median	33.8	30.9
75th centile	35.9	32.7
Max	56.5	38.4
p value[a]	0.18	0.02

[a] p-value comparing participants with and without asthma using Kruskall and Wallis test for pollutants.

Table S5: Associations between air pollution and lung function per one increase of one interquartile range in air pollutants levels (5.2 µg/m^3 for NO_2 and 3.0 µg/m^3 for PM_{10}) estimated using matching building.

	FEV_1% predict		FVC % predict	
	Beta (CI 95%)	P	Beta (CI 95%)	p
Model 0				
a- Annual mean NO_2	-1.13 (-2.70, 0.44)	0.16	-0.39 (-1.82, 1.04)	0.60
b- Annual mean PM_{10}	-1.11 (-3.32, 1.10)	0.33	0.62 (-1.40, 2.64)	0.55
c- NO_2 lag 0	0.08 (-1.57, 1.73)	0.92	-0.29 (-1.76, 1.18)	0.70
d- PM_{10} lag 0	-0.92 (-2.98, 1.14)	0.38	-0.32 (-3.26, -0.16)	0.74
Model 1				
a- Annual mean NO_2	-1.65 (-3.34, 0.04)	0.05	-1.71 (-3.26, -0.16)	0.03
b- Annual mean PM_{10}	-3.95 (-7.09, -0.81)	0.01	-3.99 (-6.87, -1.11)	0.01
Model 2				
a- Annual mean NO_2	-1.62 (-3.31, 0.07)	0.06	-1.69 (-3.14, -0.14)	0.03
NO_2 lag 0	-0.61 (-2.39, 1.17)	0.50	-0.88 (-2.49, 0.73)	0.28
b- Annual mean PM_{10}	-3.72 (-6.88, -0.56)	0.02	-3.86 (-6.76, -0.96)	0.01
PM_{10} lag 0	-1.08 (-3.22, 1.06)	0.32	-0.67 (-2.65, 1.31)	0.51

Model 0: Crude (n=354 for model a and b, n=343 for model c and n=349 for model d)

Model 1: Adjusted on sex, age, BMI, active and passive smoking, occupational group, allergic sensitization, survey and asthma (n= 321)

Model 2: Further adjustment on the pollutant level at lag 0[†] (n=310 for model a and n=316 for model b)

Table S6: Associations between air pollution and lung function per one increase of one interquartile range in air pollutants levels (5.2 μg/m^3 for NO_2 and 3.0 μg/m^3 for PM_{10}) stratified by asthma status.

	FEV1 % predicted						FVC% predicted					
	Asthmatics			Non asthmatics			Asthmatics			Non asthmatics		
	Beta	(CI95%)	p	Beta	(CI95%)	p	Beta	(CI95%)	p	Beta	(CI95%)	p
**NO_2**												
A. Building matching	-0.78	(-3.86, 2.31)	0.62	-1.89	(-3.85, 0.07)	0.06	-2.29	(-4.74, 0.15)	0.07	-1.89	(-3.78, 0.00)	0.05
B. NavTEQ	-0.46	(-3.03, 2.12)	0.72	-1.86	(-3.49, -0.24)	0.02	-0.86	(-2.93, 1.20)	0.41	-2.08	(-3.65, -0.52)	0.01
C. Google Map	0.14	(-2.21, 2.49)	0.90	-1.33	(-2.89, 0.24)	0.10	-0.44	(-2.33, 1.44)	0.64	-1.39	(-2.90, 0.12)	0.07
D. Multimap	-0.50	(-2.61, 1.60)	0.64	-1.19	(-2.59, 0.22)	0.10	-0.97	(-2.63, 0.69)	0.25	-1.48	(-2.83, -0.12)	0.03
**PM_{10}**												
A. Building matching	-3.10	(-9.41, 3.21)	0.33	-3.30	(-6.92, 0.31)	0.07	-4.19	(-9.23, 0.84)	0.10	-3.75	(-7.27, -0.22)	0.04
B. NavTEQ	-2.40	(-8.19, 3.38)	0.41	-2.51	(-5.62, 0.59)	0.11	-1.54	(-6.26, 3.17)	0.52	-3.18	(-6.21, -0.16)	0.04
C. Google Map	-1.03	(-6.89, 4.83)	0.73	-1.67	(-4.73, 1.39)	0.28	-1.35	(-6.11, 3.41)	0.57	-2.18	(-5.17, 0.81)	0.15
D. Multimap	-2.21	(-7.08, 2.66)	0.37	-1.50	(-4.31, 1.31)	0.29	-2.52	(-6.41, 1.38)	0.20	-2.32	(-5.06, 0.42)	0.10

Adjusted for: sex, age, BMI, active smoking, ETS, occupational group, atopy, level of pollutant exposure the day of examination, study and ICS for asthmatics only

NO_2 analyses include 79 participants with asthma and 231 without asthma, PM_{10} analyses include 79 with asthma and 237 without asthma.

Table S7: Associations between air pollution and lung function per one increase of one interquartile range in air pollutants levels (5.2 μg/m^3 for NO_2 and 3.0 μg/m^3 for PM_{10}) stratified by study.

	FEV1 % predicted						FVC% predicted					
	ECRHS			EGEA			ECRHS			EGEA		
	Beta	(CI95%)	p	Beta	(CI95%)	p	Beta	(CI95%)	p	Beta	(CI95%)	p
**NO_2**												
A. Building matching	-1.30	(-3.51, 0.92)	0.25	-2.59	(-5.34, 0.17)	0.06	-1.89	(-3.97, 0.18)	0.07	-1.88	(-4.24, 0.48)	0.12
B. NavTEQ	-2.09	(-3.98, -0.20)	0.03	-1.30	(-3.46, 0.86)	0.23	-2.14	(-3.92, -0.36)	0.02	-1.15	(-2.99, 0.70)	0.22
C. Google Map	-1.14	(-2.99, 0.82)	0.23	-0.86	(-2.83, 1.11)	0.39	-1.54	(-3.28, 0.20)	0.08	-0.52	(-2.20, 1.16)	0.54
D. Multimap	-1.36	(-2.96, 0.23)	0.09	-0.75	(-2.52, 1.02)	0.40	-1.55	(-3.05, -0.05)	0.04	-0.76	(-2.26, 0.75)	0.32
**PM_{10}**												
A. Building matching	-2.43	(-7.07, 2.21)	0.30	-5.42	(-10.03, -0.80)	0.02	-3.22	(-7.64, 1.20)	0.15	-4.73	(-8.75, -0.71)	0.02
B. NavTEQ	-3.08	(-7.19, 1.04)	0.14	-3.14	(-7.13, 0.85)	0.12	-2.73	(-6.66, 1.20)	0.17	-3.09	(-6.56, 0.38)	0.08
C. Google Map	-1.29	(-5.28, 2.69)	0.52	-2.77	(-6.82, 1.29)	0.18	-1.93	(-5.73, 1.86)	0.32	-2.39	(-5.94, 1.16)	0.18
D. Multimap	-1.76	(-5.25, 1.73)	0.32	-2.40	(-5.98, 1.19)	0.19	-1.91	(-5.25, 1.41)	0.26	-2.62	(-5.74, 0.49)	0.10

Adjusted for: sex, age, BMI, active smoking, ETS, occupational group, atopy, level of pollutant exposure the day of examination and asthma

NO_2 analyses include 169 ECHRS participants and 141 EGEA participants, PM_{10} analyses include 176 ECHRS participants and 140 EGEA participants.

Table S8: Associations between air pollution and lung function per one increase of one interquartile range in air pollutants levels (5.2 μg/m^3 for NO_2 and 3.0 μg/m^3 for PM_{10}), with 12-months back-extrapolated air pollution estimates, without back-extrapolation (as estimated by the SIRANE 2004 model for NO2 and the SIRANE 2008 model for PM10) and with 24-month back-extrapolated air pollution estimates (12 months before and after lung function testing)

	FEV_1% predict		FVC % predict	
	Beta (CI 95%)	P	Beta (CI 95%)	p
**12-month average exposure**[a]				
Annual mean NO_2	-1.00 (-2.59, 0.59)	0.22	-1.08 (-2.50, 0.34)	0.13
Annual mean PM_{10}	-3.15 (-6.15, -0.14)	0.04	-2.97 (-5.64, -0.29)	0.03
**No back-extrapolation**[b]				
Annual mean NO_2	-1.29 (-2.91, 0.33)	0.12	-1.37 (-2.86, 0.12)	0.07
Annual mean PM_{10}	-2.50 (-7.31, 2.31)	0.31	-2.98 (-7.41, 1.45)	0.19
**24-month average exposure**[c]				
Annual mean NO_2	-1.33 (-2.96, 0.29)	0.11	-1.41 (-2.91, 0.09)	0.06
Annual mean PM_{10}	-2.94 (-6.68, 0.79)	0.12	-3.40 (-6.84, 0.03)	0.05

All estimates adjusted for sex, age, BMI, active smoking, ETS, occupational group, atopy study and asthma (Model 2)

[a] Using the 12-month back-extrapolated air pollution estimates (results reported in the article)

[b] Using air pollution concentration estimated by the SIRANE model without back-extrapolation

[c] Using the 24-month back-extrapolated air pollution estimates (12 months before and after lung function testing)

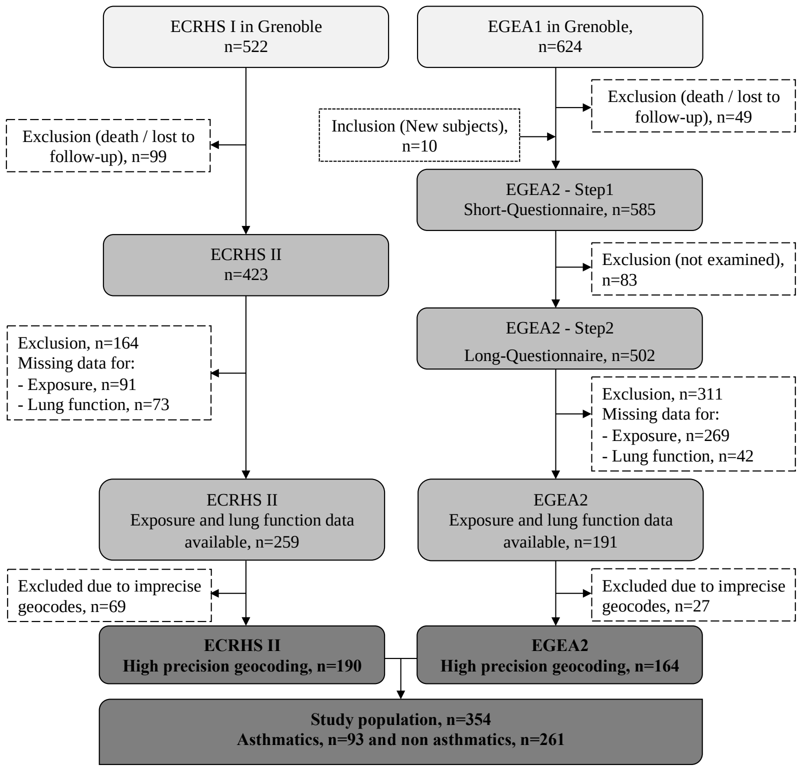

Figure S1: Description of the study population

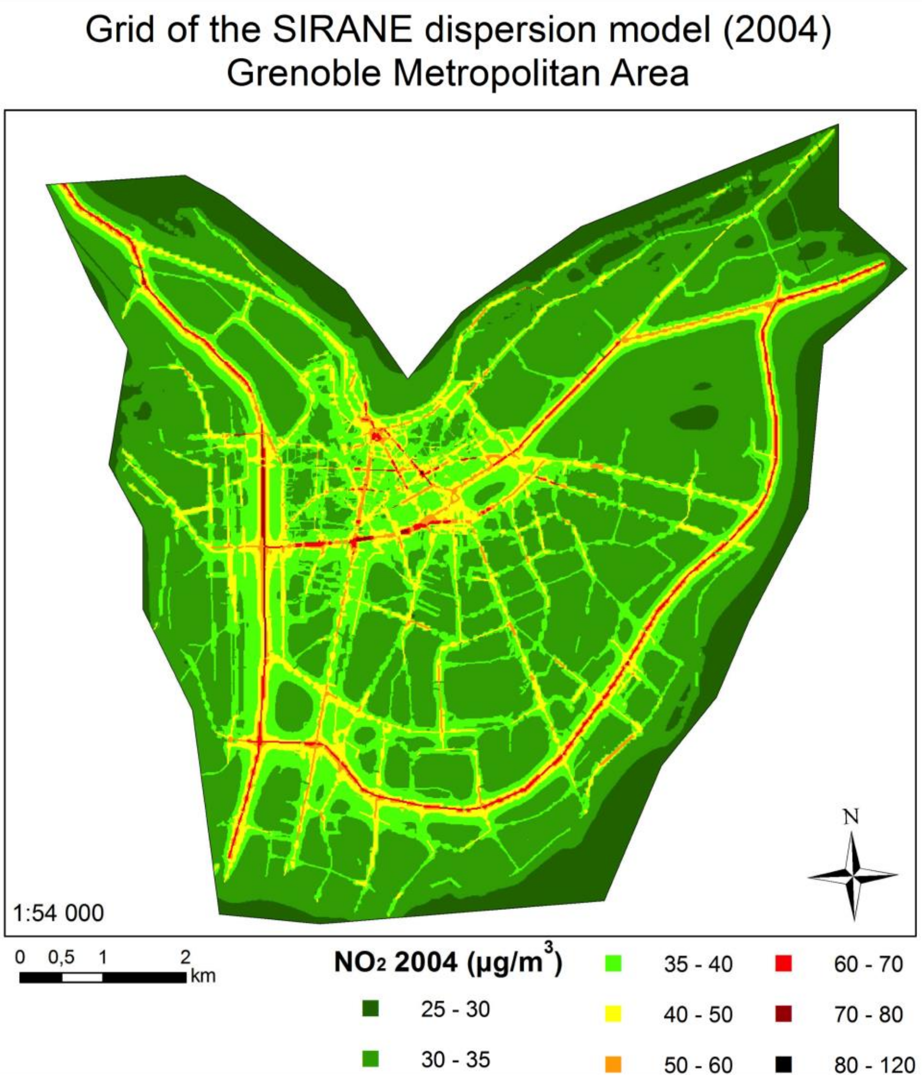

Figure S2: Mean annual NO_2 concentration estimated by the SIRANE dispersion model in 2004 in Grenoble, France.

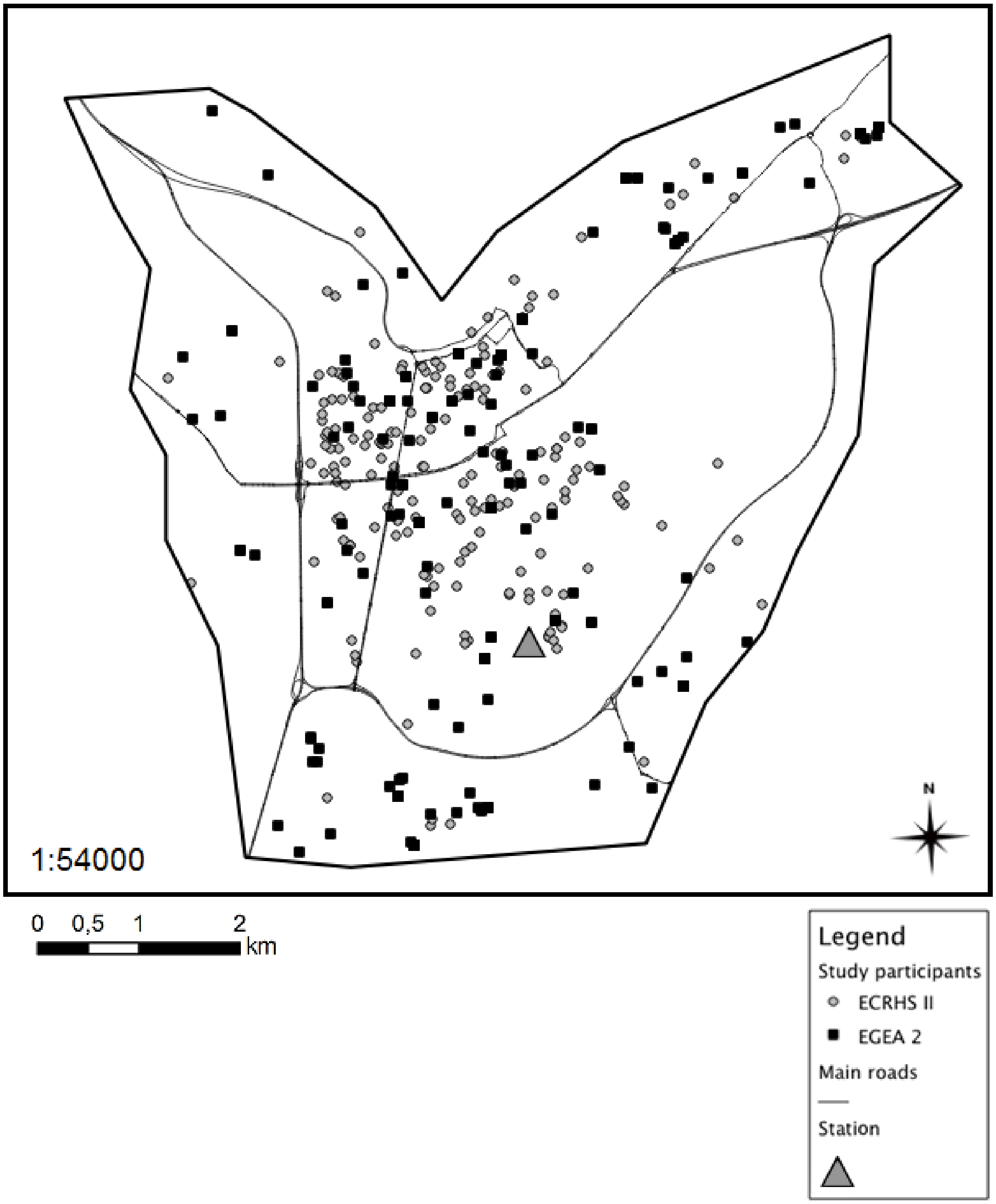

Figure S3: Map showing the participant's distribution in the Grenoble area, taking into account the study they come from. Station refers to the Villeneuve les Frênes background fixed monitoring station.
